# Lord Lister: XII. His Triumph, Complete and Universal

**Published:** 1918-04-13

**Authors:** 


### LORD LISTER.

### XII. HIS TRIUMPH, COMPLETE AND UNIVERSAL.

Lister's removal to London produced no sudden or even rapid conversion of the London surgeons to his system. King's College Hospital was a small hospital, with a small medical school of students who, like other medical students, were chiefly concerned in preparing for their examinations, and were not slow to appreciate that an advocacy of Listerism, or even a familiarity with it, would be likely to be fatal to their success in examinations conducted by men who were steeped in prejudice against the antiseptic system. From his students, therefore, Lister received no enthusiasm and little sympathy. But in the meantime the world was moving on. Listerism was adopted everywhere except in London, and London surgeons began to recognise with uneasiness that they were being left behind, and that the world was going on without them. The writer of this notice was from the beginning of the 'seventies a student or on the resident staff of one of the largest of the London hospitals, and during the seven years of his studies at that hospital no allusion of any kind was ever made to Listerism or to the antiseptic system, or to antiseptics in any surgical lecture or in any surgical teaching at that hospital. Only one of the surgeons, a Scotchman, adopted a practice that purported to be that of Lister, and the results achieved by this solitary disciple were no better than those attained by his colleagues, who still operated in their filthy old operating coats and after the filthy old methods. Still, the world moved on, and London surgeons found themselves opposed to the practice, not of Edinburgh alone, but of the whole world. *Securus judicat orbis terrarum.* Doubts and misgivings at last sapped their confidence in their own methods. One after another they yielded to the inevitable and adopted Lister's practice, and long before he retired from the professorship at King's College he witnessed its almost universal prevalence in the last stronghold of dirt, pyæmia, and septicæmia. Long before he died his triumph was complete and universal.

Disappointing and mortifying as his first experiences in London were, it was not very long before his removal to the metropolis resulted in his emergence into a larger life of wider and more numerous interests than could ever have been attained in a provincial city, even one so famous for intellectual activity and achievement as Edinburgh. In London the societies for the discussion of matters of medical interest were numerous, and afforded him many opportunities for spreading the light, and for bringing before the medical public the results of his new researches, for he still continued those scientific experiments that formed the basis of his practice. He was elected to the Council of the Royal College of Surgeons; and might have been its President if he had chosen. He was much concerned in the reconstitution of the University of London. He bestirred himself to found an Institute on the lines or for the purpose of the Pasteur Institute in France, for the study of infective diseases. This was eventually started, Lister having taken the lead in bringing to the country and the Government a knowledge of its desirability, and, indeed, of its necessity in any civilised State. Lister proposed that it should be called the Jenner Institute, in commemoration of the discoverer of vaccination, and his name was, in fact, given to the Institute, but the name was eventually changed on account of the opposition of a commercial undertaking, called the Jenner Institute for Calf Lymph. The title eventually given to the Institute was that of the Lister Institute of Preventive Medicine. It languished for many years from lack of interest and lack of funds, until at length Lord Iveagh bestowed upon it the princely gift of a quarter of a million pounds, and started it upon that career of active beneficence that has enabled it to benefit every man, woman, and child throughout this realm, and to advance our knowledge of disease and our means of preventing it so as to benefit humanity at large. Lister was the first Chairman of the Governing Body, and eventually the President of the Institute which worthily commemorates his name.

In 1892 Lister retired under the age-limit from his professorship at King's College, and in the following year relinquished his beds at King's College Hospital and at the same time his private practice. Henceforth he was a man of leisure, but he was as far as ever from being an idle man. He not only continued the scientific experiments, which he had never abandoned even in his busiest days, but found his time more and more occupied as a public man. He became President of the Royal Society and President of the British Association, the two highest honours that any scientific man in this country can aspire to. He was sought for to give addresses, to read papers, to represent his country at Congresses and Committees, to act as Chairman of this and President of that, until at length his fame and his immeasurable services to humanity, long known in every corner of the civilised world, celebrated and acclaimed in every country of every continent on the face of the globe, at last compelled a tardy, unwilling, and most inadequate recognition from even the politicians of his own country. That politicians should be utterly ignorant of science, and supremely contemptuous of that of which they are ignorant is the accepted and intended result of the public-school and University education in which practically all professional politicians have been brought up. They look upon the man of science, if indeed they allow their thoughts to rest at all upon such an insignificant being, much as the ordinary middle-class householder looks upon the scavenger who removes the contents of his dustbin; that is to say, as a useful person, who dabbles in unsavoury material, whose services we are glad to avail ourselves of, but who belongs to an

☛ Previous articles appeared Jan. 26, Feb. 2, 9, 16, 23, March 2, 9, 16, 23, 30, and April 6, p. 9.

**LORD LISTER**—(*continued*).

altogether inferior social scale, who is not a person to associate with, and who must be kept strictly in his place if by any strange chance he should show signs of desiring recognition as anything but a scavenger. It was something of a scandal to the politicians of the end of the last century that one of their own body, and a leader among them no less prominent than Lord Salisbury, should be credited by rumour with a taste for dabbling in electricity or chemistry, the politicians were not quite sure which. Nor would it have mattered, for few of these could have told the difference between them, or whether there is any difference between them. However, they are both scientific subjects, and therefore unfit to engage the attention of a being so superior as a politician, and if Lord Salisbury had not been so great a lord, he would have been in danger of losing caste by his experiments, whatever they were.

But even Lord Salisbury, great politician as he was, and wielding as he did an autocratic and undisputed authority over his party, even Lord Salisbury never thought until very late in Lister's life of bestowing any official recognition or honour upon the man who was undoubtedly one of the greatest and the best known, and most universally acclaimed Englishmen of his generation. At last, however, the claim of the ignoble scientist to recognition became so manifest that to withhold it longer would have been a scandal. It was, indeed, already a scandal that it had been so long withheld. Lord Salisbury, therefore, in 1897, when Lister was seventy years old, at long length offered him—what? The very lowest rank in all the peerage. It was ridiculously and scandalously inadequate, but it was something. It was tardy, it was grudging, it was less than is given to a party hack who has outlived his usefulness in the Commons and must be got rid of by being kicked upstairs into the House of Lords; but it was something. Lord Salisbury was careful, however, to make it clear that even the barony bestowed upon the man for whom a dukedom would have been an inadequate reward, was not bestowed upon the surgeon. That unimportant part of Lister's activities was ignored. The gilded chamber of the peers would have been desecrated by the entry among its noble occupants of a mere doctor as such; so a barony was offered to Lister in consideration of the extraordinary services that he had rendered to science, not of the extraordinary benefits that he had conferred upon mankind by his application of these scientific principles to surgery and medicine. Such was the power of the principle of caste at the end of the nineteenth century. Such was the ingrained prejudice of a comparatively enlightened politician who had been brought up in the common tradition of the public school and the University of Oxford.

Lister's declining years were marred by sorrow and ill-health. The severest penalty of advancing years is the loneliness that so often comes with them. He who lives to a great age of necessity outlives his friendships, for friends made in early life die off, and intimate friendships are seldom contracted after middle age. His great grief came in 1893 with the death of his wife, his most devoted companion, assistant, amanuensis, and colleague. After this, life lost its savour. He contracted his social circle. He ceased to entertain. But he continued his public activities, and he did not cease his researches and experiments. At length, in 1903, at the age of seventy-six, he had a serious attack of illness of an obscure character, which debilitated both his bodily and his mental powers, and thenceforth his active life was over. Little by little his powers ebbed away. His sight failed till he could no longer read or write. His mental energy failed, so that he dreaded the visits of even his nearest and dearest relatives. His bodily power failed, so that he was confined, at first to the house, and at last to bed; and at length he became unconscious, and so slipped through the gloomy portal on the 10th of February, 1912. So ended Joseph Lister, full of years and honours, the greatest benefactor mankind has ever had.

(*Concluded.*)

